# Codabench: Flexible, easy-to-use, and reproducible meta-benchmark platform

**DOI:** 10.1016/j.patter.2022.100543

**Published:** 2022-06-24

**Authors:** Zhen Xu, Sergio Escalera, Adrien Pavão, Magali Richard, Wei-Wei Tu, Quanming Yao, Huan Zhao, Isabelle Guyon

**Affiliations:** 1 4Paradigm, Beijing 100085, China; 2 Computer Vision Center, Universitat de Barcelona, 08007 Barcelona, Spain; 3 LISN/CNRS/INRIA, University Paris-Saclay, 91190 Gif-sur-Yvette, France; 4 University Grenoble Alpes, CNRS, UMR 5525, VetAgro Sup, Grenoble INP, TIMC, 38000 Grenoble, France; 5 Tsinghua University, Beijing 100084, China; 6 ChaLearn, Berkeley, CA, USA

**Keywords:** machine learning, data science, benchmark platform, reproducibility, competitions

## Abstract

Obtaining a standardized benchmark of computational methods is a major issue in data-science communities. Dedicated frameworks enabling fair benchmarking in a unified environment are yet to be developed. Here, we introduce Codabench, a meta-benchmark platform that is open sourced and community driven for benchmarking algorithms or software agents versus datasets or tasks. A public instance of Codabench is open to everyone free of charge and allows benchmark organizers to fairly compare submissions under the same setting (software, hardware, data, algorithms), with custom protocols and data formats. Codabench has unique features facilitating easy organization of flexible and reproducible benchmarks, such as the possibility of reusing templates of benchmarks and supplying compute resources on demand. Codabench has been used internally and externally on various applications, receiving more than 130 users and 2,500 submissions. As illustrative use cases, we introduce four diverse benchmarks covering graph machine learning, cancer heterogeneity, clinical diagnosis, and reinforcement learning.

## Highlights

• Codabench facilitates flexible, easy, and reproducible benchmarking
• Organizers can customize benchmark design and submission format
• Organizers may host their own platform instance or use the public instance
• Four use cases in diverse domains are introduced to demonstrate the key features

## The bigger picture

In almost all communities working on data science, researchers face increasingly severe issues of reproducibility and fair comparison. Researchers work on their own version of hardware/software environment, code, and data, and consequently, the published results are hardly comparable. We introduce Codabench, a meta-benchmark platform, that is capable of flexible and easy benchmarking and supports reproducibility. Codabench is an important step toward benchmarking and reproducible research. It has been used in various communities including graph machine learning, cancer heterogeneity, clinical diagnosis, and reinforcement learning. Codabench is ready to help trendy research, e.g., artificial intelligence (AI) for science and data-centric AI.

## Teaser

Fair and flexible benchmarking is a common issue in data-science communities. We develop the Codabench platform for flexible, easy, and reproducible benchmarking. It is open sourced and community driven. With Codabench, we are able to fairly and easily compare algorithms as well as datasets under diverse protocols. The reproducibility is also guaranteed.

## Introduction

The methodology of unbiased algorithm evaluation is crucial for machine learning and has recently received renewed attention in all data-science scientific communities. Often, researchers have difficulties understanding which dataset to choose for a fair evaluation, with which metrics, under which software/hardware configurations, and on which platforms. The concept of a benchmark itself is not well standardized and includes many settings. For instance, the following may be referred to as a benchmark: a set of datasets, a set of artificial tasks, a set of algorithms, one or several dataset(s) coupled with reference baseline algorithms, a package for fast prototyping algorithms for a specific task, or a hub for compilation of related algorithm implementations. In addition, many benchmarks often integrate new progress by manual verification instead of automatic submission and execution, which delays the benchmark update and requires extra human efforts.

Typical examples of existing frameworks addressing such needs are inventoried in Table 1, including competition platforms, repository hubs, and domain-specific benchmarks. Firstly, competition platforms focus on the participants and provide limited support for organizing general tasks. Famous platforms like Kaggle (https://www.kaggle.com/), Tianchi (https://tianchi.aliyun.com/), and CodaLab (https://codalab.lisn.upsaclay.fr/) organize many data-science challenges, attracting a large number of participants. However, the platform providers retain some control: the organizers do not have full flexibility and control over their competitions. Thus, the experience for organizers is not enjoyable. A comparison between competitions and benchmarks is given in Table S1. Secondly, repository hubs such as UCI repository (https://archive.ics.uci.edu/ml), OpenML,^1^ and PapersWithCode (https://paperswithcode.com/) also play an important role for benchmarks and research. They collect large numbers of datasets, methods, and results from academic papers, but reproducibility by running code in given containers (or similar ways) is not guaranteed. Besides the above-mentioned platforms, many domain-specific benchmarks exist, e.g., DAWNBench^2^ and KITTI Benchmark Suite.^3^ These benchmarks usually focus on a couple of closely related tasks but are not designed to host general benchmarks. In addition, they require repetitive efforts to develop and maintain, which is not always affordable by data-science teams. Thus, to facilitate benchmarking, we need a platform to allow users to flexibly and easily create benchmarks with custom evaluation protocols and custom data formats, with execution in a controlled, reproducible environment, that is totally free and open sourced.

**Table 1:** Comparison of various reproducible science platforms

Platform	Flexibility			Easy to use				Reproducibility
	Bundle	Result/code submit	Dataset submit	Easy creation	Open source/free	API access	Compute queue	
Kaggle	✗	✓	✗	✓	✗	✓	✓	✓
Tianchi	✗	✓	✗	✓	✗	✗	✓	✓
CodaLab	✓	✓	✗	✓	✓	✗	✓	✓
UCI	✗	✗	✓	✗	✓	✗	✗	✓
OpenML	✗	✓	✓	✓	✓	✓	✗	✓
PapersWithCode	✗	✓	✗	✓	✓	✗	✗	✓
DAWNBench	✗	✓	✗	✗	✓	✗	✗	✓
Codabench	✓	✓	✓	✓	✓	✓	✓	✓

Different features are introduced in the section key features of Codabench. Bundle means whether a wrap up is provided for a benchmark such that we could reuse or share. Result/code/dataset submit means whether different submissions are supported to enable flexible tasks. Compute queue means where public or private computation resources could be provided or linked for convenient deployment.

**Figure 1:**
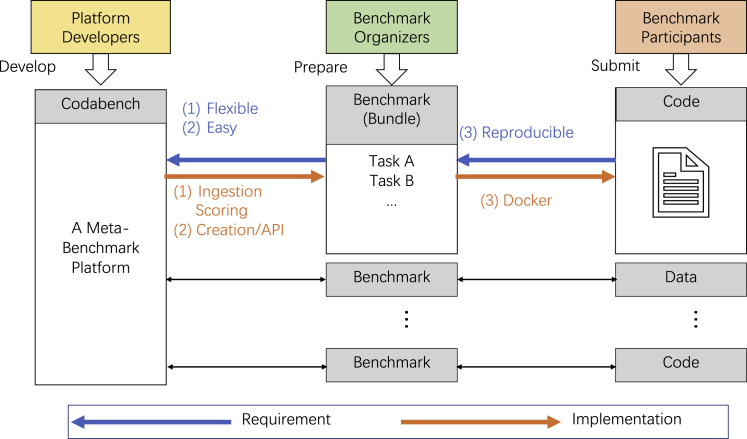
Overview of Codabench A meta-benchmark platform has three types of contributors: platform developers (in yellow), benchmark organizers (in green), and benchmark participants (in red). Codabench is at the meta level to support diverse benchmarks. Each benchmark is implemented by a benchmark bundle that contains one or more tasks.

To answer these unmet needs, we developed Codabench, a meta-benchmark platform (Figure 1). A meta-benchmark platform is designed to support general-purpose benchmarks and to facilitate the organization and usage of benchmarks. Codabench takes into account three types of contributors: benchmark participants, benchmark organizers, and platform developers. Benchmark participants submit to different benchmarks, which are prepared and owned by different benchmark organizers. Reproducibility is required at this stage for fair benchmarking. Platform developers contribute different features to Codabench to support diverse benchmarks instead of one specific benchmark, i.e., Codabench is at the meta level of benchmarks. Flexibility and easiness to organize and use benchmarks are thus required at this stage. Codabench realizes these features by implementing an ingestion/scoring programming paradigm, supporting multiple benchmark creation methods and application programming interface (API) access, and using Docker to guarantee reproducibility. Codabench has received over 130 users and 2,500 submissions on 100 tasks including automated machine learning (AutoML),^4^ graph machine learning,^5^ reinforcement learning (RL),^6^ detecting cancer heterogeneity, and training clinicians (https://cancer-heterogeneity.github.io/). Multiple illustrative use cases are introduced. Codabench is an important step toward reproducible research and should meet the interest of all areas of data science.

### Method: Design of Codabench

**Figure 2:**
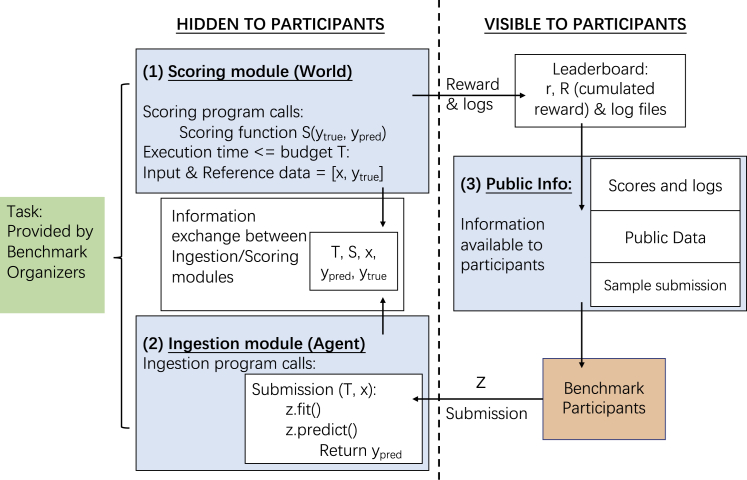
Operational Codabench workflow Left: task module specified by the benchmark organizers, executed on the platform. Right: web interface with participants permitted to make submissions and retrieve results. Numerated blocks are specified by the benchmark organizers. They include (1) a scoring module, (2) an ingestion module, (3) and public information. An intermediate block also exists for information exchange of time budget, scoring, input data, ground-truth data, and predictions. Red bottom-right block: participant prepares a submission “z” uploaded to the platform. The submission is then executed by the ingestion program. The role of the scoring program is to produce scores that are then displayed on the leaderboard.

Codabench is a meta-benchmark platform that provides a flexible, easy-to-use, and reproducible benchmarking service that is publicly and freely available for everyone. In Codabench, benchmarks are implemented by benchmark bundles, which contain one or several tasks. The concept of a task is newly introduced, which is the minimal unit for composing a benchmark (bundle). A task consists of an “ingestion module” (including an ingestion program and input data), a “scoring module” (including a scoring program and reference data, invisible to the participant’s submission), a baseline solution with sample data, and meta-data information if needed. Tasks in Codabench may be programmed in any programming language in any custom way and are run in a docker specified by organizers. Figure 2 provides a detailed description of Codabench internal interaction logistics.

Take supervised-learning tasks as an example. A typical usage is that benchmark participants submit a class (e.g., a Python object) “z”, with 2 methods, z.fit and z.predict, similarly to scikit-learn objects.^7^ The ingestion program reads data, calls z.fit with labeled training data and z.predict with unlabeled test data (labeled training data and unlabeled test data being part of the so-called “input data”), then outputs predictions. The scoring program reads the predictions and evaluates them based on custom scoring metric(s) using the test labels (called “reference data”). Other application usages are possible, including transposed benchmarks, where datasets are submitted by participants instead of algorithms, while the organizers supply a set of algorithms, and RL benchmarks, where the ingestion program plays the role of an agent wrapping around the submission of the participant and interacting with a world (scoring program) in a specific way.

The reader is referred to the Codabench official repository (https://github.com/codalab/codabench/), where the code and complete documentation are found. In the supplemental information, we also include instructions and references to get started. To use the public instance of Codabench, please visit the Codabench website. To test and install locally, the instructions are given in the README file of the official repository. The Codabench code is released under an Apache 2.0 license.

## Results

### Key features of Codabench

Codabench is task oriented. Using tasks, the organizers have the flexibility of implementing any benchmark protocol, with any dataset format and API, or even using data-generating models, allowing them to organize RL challenges. In this section, we introduce the key features of Codabench contributing to its flexibility, ease, and reproducibility, as shown in Figure 1. Codabench also supports custom leaderboards and has full documentation of usage.

#### Flexibility

Codabench supports flexible benchmark types including results submission, code submission, and even dataset submission. Benchmarks on Codabench are organized by bundles containing all the information of a benchmark.

Bundle (hosting a benchmark): a benchmark bundle is a zip file containing all necessary constituents of a benchmark, including tasks, documentation, and configuration settings (such as leaderboard settings). A Codabench bundle may include a single or multiple tasks. A classical benchmark is usually single task while AutoML,^4^ transfer learning,^8^ and meta learning^9^ benchmarks are multitask.

Results or code submission: “classic” Codabench benchmarks are either with result or code submission. On one hand, result submissions are used when organizers wish that participants use their own computational resources. In supervised-learning competitions, participants would supply, e.g., predictions of output values on some test datasets. Other types of results may be supplied, for instance, high-resolution images in a hyper-resolution challenge for which inputs are low-resolution images. On the other hand, if the organizers wish to run all algorithms in a uniform manner on the platform, Codabench allows the participants to make code submissions. The submitted software is run in a docker supplied by the organizers, either on the default compute worker or on compute workers supplied by the organizers. This code-submission design allows organizers to provide suitable computational resources (e.g., GPUs) and improve reproducibility.

Dataset submission: the role of the dataset and algorithm can be transposed with Codabench to facilitate data-centric artificial intelligence (AI) (https://datacentricai.org/), which is a trending research topic that cares about the quality and usage of data. In a classic benchmark, organizers provide datasets, and participants submit algorithms. In a transposed benchmark, participants submit datasets, and organizers provide reference algorithms. A classic benchmark will have a leaderboard with datasets in columns that grows by adding more lines as algorithm submissions are made. In a transposed dataset-submission benchmark, the leaderboard will have algorithms in columns, and lines are added as more datasets are submitted. With Codabench’s transposed benchmark, it is easier to try different data-augmentation and -processing methods with fixed algorithms as test cases.

#### Easy to use

To facilitate an easiness of using the system, we provided several tools to help the benchmark organizers create a benchmark. A platform editor is provided to develop a benchmark, which provides simple user interfaces to prepare data, code, and other configurations. As a second option, the user can upload a locally prepared benchmark bundle to facilitate local debugging and testing. Once uploaded, a benchmark can further be modified using the platform editor. An existing benchmark can be saved as another bundle, which facilitates sharing and portability. Similar benchmark bundles can be easily prepared with shared template bundles. Codabench is open sourced and free to use.

APIs to external clients: we provide APIs for interacting with the platform, including “robot” submissions via command lines, without going through the regular Codabench web interface, and this is likewise a programmatic way of recuperating results directly without going through the leaderboard.

Dedicated computing queues: the public instance of Codabench provides default compute workers. Organizers can also create a dedicated job queue and connect it to their own CPU or GPU compute workers.

#### Reproducibility

Codabench makes extensive use of dockers (https://www.docker.com/) to guarantee reproducibility. Benchmark organizers specify the docker image by providing its docker hub name and tag. Docker wraps all the software dependencies into a lightweight virtual image. Once a docker is provided by the benchmark organizer, the program can be run inside a docker that contains exactly the same installed packages. This docker will be pulled every time a benchmark’s program is executed. Different benchmarks could use different dockers, which are usually provided by organizers. We also provide a default docker for more general benchmarks’ usage or people who are not familiar with dockers.

#### Other features

Custom leaderboard: to better facilitate benchmarks, the leaderboard is fully customizable and can handle multiple datasets and multiple custom scoring functions. We provide multiple ways to display submissions (best per participant, multiple submissions per participant, etc.), and the leaderboard can flexibly rank submissions by average score, per task, per submetric of a certain task, etc.

Documentation: the documentation (https://github.com/codalab/codabench/wiki) provides detailed help for different types of contributors. For benchmark participants, we provide instructions to join and submit to a benchmark. For benchmark organizers, we provide annotated instructions for organizing benchmarks. Several benchmark-bundle templates, from simple to advanced, are also available to ease the technical aspects of building a benchmark and to let people concentrate on scientific aspects of the benchmark. For platform developers, we explain more technical specifications on technology stack and provide ways to integrate to the project. Platform developers are contacted via the GitHub issues and pull requests to solve issues encountered in daily usage.

### Use cases of Codabench

**Figure 3:**
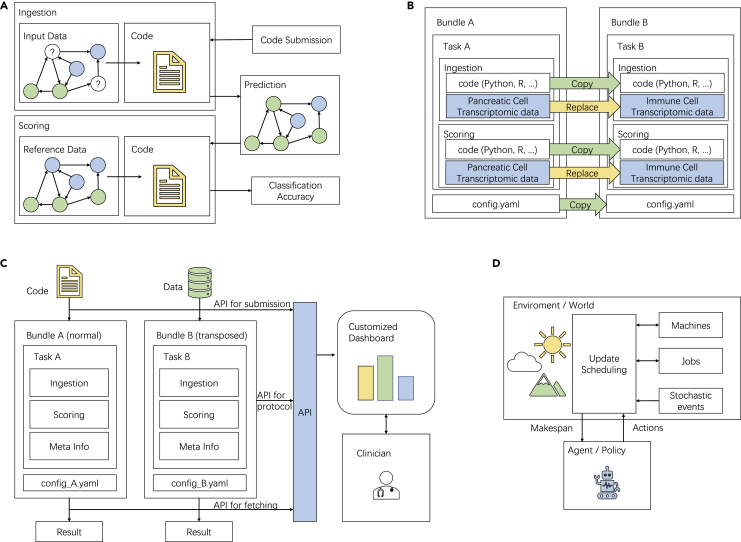
Use-case illustrations Four use cases are introduced: (A) AutoGraph, (B) DECONbench, (C) COMETH, and (D) job scheduling. The use-case details are introduced in the section Use cases of Codabench.

Codabench has been used not only internally at 4Paradigm and LISN Lab for tasks of AutoML,^4^ graph machine learning,^5^ RL,^6^ speech recognition,^10^ and weakly supervised learning^11^ but also externally by University Grenoble Alphes for hosting scientific benchmarks in cancer heterogeneity and training clinicians. Codabench has received more than 130 users and 2,500 submissions distributing on various applications. In this section, we introduce 4 use cases of Codabench, aiming at demonstrating different Codabench features and capabilities. A visual illustration is given in Figure 3.

#### Use case 1: AutoGraph benchmark

In this section, we introduce automated graph machine learning (AutoGraph) benchmark, which targets automated node classification methods on diverse dataset scenarios. With this use case, we show a set of fundamental features of Codabench: (1) the code submission mode, (2) reproducibility guaranteed by docker, (3) flexible benchmark-bundle configuration with multiple tasks, and (4) customizable computational resources.

Background: graph machine learning has been a very hot topic due to the ubiquity of graph-structured data, e.g., social networks,^12^ molecule graphs,^13^ knowledge graphs,^14^ etc. Typical tasks of graph data include node level (node classification), edge level (link prediction), and graph level (graph regression/classification). The task of our benchmark here is node classification, i.e., given a graph where some nodes are labeled and the rest are unlabeled, we want to predict the classes of the unlabeled nodes. In addition, we require the algorithm to perform well on a set of datasets instead of just one dataset. This leads to automated-graph-machine-learning problem, which we call AutoGraph.

Implementation: the AutoGraph benchmark is a typical code-submission use case. It focuses on AutoML methods,^4^ which requires more than one dataset to be evaluated together. A Codabench bundle is, by design, flexible with multiple tasks, each of which contains a separate dataset. We also provide a docker hosted on DockerHub, which could be pulled automatically by the Codabench platform to run each algorithm submission and be used for researchers’ local development. Every time a new method is uploaded, a new docker container instance will be called to independently run the method for each dataset. In this way, we make sure every algorithm is fairly run under the same setting and that the whole process can be fully reproduced on other machines. Codabench is designed to adapt to any docker-enabled computational resource (local machine, cluster server, cloud machines, etc.). We currently host the AutoGraph benchmark on Codabench with free computational resources thanks to Google’s sponsorship, encouraging everyone to contribute. Besides, the datasets are also available to the public for local usage and further benchmarking on GitHub (https://github.com/AutoML-Research/AutoGraph-KDDCup2020) and Kaggle. We uploaded the solutions of the winners of the challenge as baselines. Since the benchmark datasets are already released, users can also run complementary experiments on their local computers and debug mode easily, thus making progress more rapidly. With the AutoGraph benchmark, we provide researchers and practitioners the possibility to showcase results in a public venue.

#### Use case 2: DECONbench benchmark

In this section, we introduce the DECONbench benchmark.^15^ DECONbench aims at benchmarking algorithms inferring tumor cellular composition from molecular data. Here, we highlight two features of Codabench: (1) flexibility of benchmark bundles (in this use case, another task and programming language R supported) and (2) reusability and portability of benchmark bundles.

Background: successful treatment of cancer is still a challenge, and this is partly due to a wide heterogeneity of tumor cellular compositions across patient population. Tumors are made up of cells with different identities and origins. Cancer cells evolving in a dynamic environment consist of aberrant non-cancerous cells, such as blood vessels or immune cells. Tumor cellular composition is difficult to observe and quantify, as it is hidden inside the bulk molecular profiles of the samples, with millions of cells present in the tumor (and not only cancer cells) contributing to the bulk recorded signals. Taking advantage of the large amount of molecular data publicly available, a wide number of supervised and unsupervised algorithms have been recently developed to estimate tumor cellular composition.^16^, ^17^, ^18^ DECONbench is a series of benchmarks dedicated to the quantification of tumor composition, focusing on estimating cell types and proportion in biological samples using multimodal molecular data. Participants have to identify an estimation of the tumor composition, i.e., a matrix of the estimated proportion of each deconvoluted cell type (rows) for each sample (columns). The discriminating metric is the mean absolute error (MAE) between prediction and ground-truth matrix. Note that the DECONbench series is optimized to run methods developed in the statistical programming language R.

Implementation: using the Codabench platform, the COMETH consortium firstly developed a benchmark for continuous evaluation of computational methods based on epigenomic data (https://www.codabench.org/competitions/174). Since we are at the same time interested in other modalities of data under similar tasks, it would be ideal to reuse previously created bundles instead of going through everything again. Thanks to the portability of the Codabench bundle design, we only need to replace the data files and adjust slightly the protocol code. All other configuration files can be reused. As a result, this first benchmark was easily cloned and extended to similar benchmarks using other types of data, e.g., all-cell-type transcriptomic data (https://www.codabench.org/competitions/147), immune-cell-type transcriptomic data (https://www.codabench.org/competitions/148), and all-cell-type multimodal transcriptomic and epigenomic data (https://www.codabench.org/competitions/237).

#### Use case 3: COMETH benchmark

In this section, we introduce the COMETH benchmark, which is motivated by real clinical application and is an exciting step toward data-centric AI. With this use case, we show that (1) Codabench supports a transposed benchmark consolidating data-centric AI and (2) the provided API interaction opens a window for other customization scenarios.

Background: when it comes to clinical application, it is often necessary for health-data scientists and clinicians to identify the most suitable existing method to be applied on a given dataset. In this case, we focus more on the data instead of algorithmic development, which aligns with data-centric AI. Usually, the clinicians do not (need to) know much about the algorithm details. Instead, they have access to newly available data and want to apply the most relevant algorithms on their new data. There is thus a need to provide an effective tool displaying the evaluation of state-of-the-art algorithms on reference datasets and enabling their application on new datasets. This will guide and facilitate the appropriate use of these algorithms by non-expert clinicians. Note that these algorithms are usually provided by benchmark organizers who are domain experts on certain tasks.

Implementation: to solve this question, the COMETH consortium developed the COMETH benchmark (https://www.codabench.org/competitions/218), a transposed challenge in which datasets should be submitted to be evaluated against existing algorithms (i.e., tasks in the Codabench design). For instance, the COMETH benchmark provides a series of recent deconvolution algorithms that are able to quantify tumor heterogeneity.^16^, ^17^, ^18^ Clinicians aiming to quantify tumor heterogeneity from molecular data can submit their dataset of interest to the COMETH benchmark and retrieve the corresponding outputs in a fully reproducible environment. To facilitate the use of this functionality by clinicians who are less familiar with data-science programming details, the COMETH benchmark has been connected to an external client displaying a user-friendly web dashboard. The external client is able to send requests to users directly on the COMETH benchmark using APIs provided by Codabench and return the generated results from all reference algorithms. This feature strongly contributes to a direct transfer of knowledge between data scientists and healthcare professionals. This design was used at a winter school for training clinicians and data scientists (https://cancer-heterogeneity.github.io/cometh.html).

#### Use case 4: Job scheduling benchmark

We lastly introduce another use case, the job scheduling benchmark, which focuses on RL and operational research. With this use case, we show that Codabench is RL friendly with the help of flexible designs of benchmark bundles.

Background: we consider the problem of dynamic job shop scheduling.^19^, ^20^, ^21^ The task is to allocate a set of jobs to a set of machines to achieve the shortest execution time, i.e., makespan. Each job has a pre-determined operation sequence to be executed on certain machines. To mimic real-life scenarios, we add stochastic machine-down events to the problem. This task is usually formulated as a sequential decision-making problem and fits easily to RL. We thus expect an agent making decisions on how to better schedule the jobs in minimal time. The reward depends on the makespan.

Implementation: as explained in the section method: design of Codabench, our design of bundles and an ingestion/scoring program makes it very natural and flexible for RL problems. We easily use the scoring program as an environment that evaluates a job schedule and returns a makespan as reward. The ingestion program serves as an agent and makes decisions on job scheduling based on the received reward.

### A concrete benchmark-bundle example

**Figure 4:**
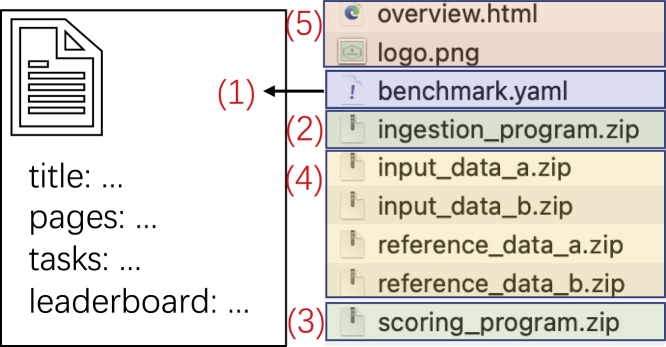
Bundle structure The details of benchmark.yaml is given in Data S1.

In this section, we provide a concrete benchmark-bundle example to show how simple it is to organize benchmarks on Codabench. A bundle consists of five parts, as in Figure 4: (1) a YAML configuration file (https://yaml.org/), (2) an ingestion program, (3) a scoring program, (4) data, and (5) additional files for description.

The ingestion program usually reads data and a participant’s submission. It calls the participant’s method on the dataset and produces predictions to a shared space. The scoring program usually reads the ingestion program’s output and evaluates with respect to (w.r.t.) ground truth according to an organizer’s customized metric. It finally writes scores to a text file, which will be read by the platform and be displayed on a leaderboard. The data contain input data (in supervised learning, they are usually X_train, y_train, and X_test) and reference data (in supervised learning, it is usually y_test). Both are zipped into separate files. The additional files are just text or figure files for organizers to provide other information, e.g., instructions, references, logo, etc. A final YAML file connects all previous parts and provides more configurations for the benchmark. A simplified YAML file is given in Data S1. It contains general configurations like title, logo image, docker image, which HTMLs are to be displayed, leaderboard configuration (e.g., which metrics will be used in the leaderboard), and tasks. Each task is by itself a complete unit for running. It contains name, ID, ingestion program, scoring program, input data, reference data.

## Discussion

Codabench is a new meta-benchmark platform for data-science communities. Codabench is compatible with diverse tasks (including supervised learning and RL) and supports result, code, and dataset submission. It is easy to use Codabench, and reproducibility is guaranteed by dockers. Codabench has a public instance free for use, deployed at Université Paris-Saclay, but can also be deployed locally with the technology stack provided in documentation. Hosting, maintaining, and further developing the platform is funded by grants and donations. As real-world scenarios, we introduce 4 benchmark use cases illustrating the flexibility, ease of use, reproducibility, and other features of Codabench. We also note that tremendous other tasks could be integrated into Codabench as well including electroencephalogram (EEG) classification, drug discovery and property prediction, and dynamic simulation for weather, traffic, fluid, etc., which are important tasks toward AI for science.

The current limitations of Codabench are mainly as follows. First, since it is relatively new, we do not have yet an active community of organizers and benchmark participants. We need more users’ feedback to polish up our user interface and documentation. Second, although supported by design, we have not yet had a distributed computation scenario where complex multi-node compute workers are used. This could enrich our benchmark template library with benchmarks for algorithm parallelization. Thirdly, although Codabench supports both code and dataset submissions, we do not currently allow users to extend the leaderboard in both directions simultaneously, i.e., it does not allow users to submit both code and datasets at the same time. This feature could largely increase the user experience of the platform. Lastly, Codabench does not yet support hardware-related benchmarks or human-in-the-loop benchmarks, which could be interesting to consider in the future.

Potentially harmful uses of Codabench could result from poor benchmark designs (e.g., no scientific question is asked by hosting a benchmark) or bad data collections (e.g., data license, data quality), as in any machine-learning project. We are working on an open-access book (to appear in 2022) on best practices for designing challenges and benchmarks including data preparation, task evaluation, etc. and for post-challenge/post-benchmark analysis.

Further works include providing more comprehensive usage templates illustrating features such as (1) splitting an algorithm workflow into submodules and scoring the effectiveness of the modules individually (e.g., with ablation or sensitivity analysis), (2) providing templates of fact sheets to extract information about algorithms (similar to datasheets for datasets but for algorithms), and (3) providing guidelines to benchmark participants to produce enriched detailed results, amenable to meta-analyses.

## Experimental procedures

### Resource availability

#### Lead contact

The lead contact is Zhen Xu (xuzhen@4paradigm.com), who is a research scientist at 4Paradigm, Beijing, China.

#### Materials availability

This study did not generate new materials.

## Data Availability

The code of Codabench is available at https://github.com/codalab/codabench. This work does not introduce new datasets. For creating benchmarks, organizers should prepare their own datasets.

## Supplemental information

Document S1. Supplemental experimental procedures, Table S1, Figure S1, and Data S1

Document S2. Article plus supplemental information

## Footnotes

Supplemental information can be found online at https://doi.org/10.1016/j.patter.2022.100543.
