# Supplementary material for: Codabench: Flexible, easy-to-use, and reproducible meta-benchmark platform
Source: Patterns (N Y). 2022 Jun 24;3(7):100543. doi: 10.1016/j.patter.2022.100543 (PMC9278500; doi:10.1016/j.patter.2022.100543)
Supplement: Document S1. Supplemental experimental procedures, Table S1, Figure S1, and Data S1 [file mmc1.pdf]

Patterns, Volume *3*

Supplemental information

Codabench: Flexible, easy-to-use, and reproducible meta-benchmark platform

Zhen Xu, Sergio Escalera, Adrien Pavão, Magali Richard, Wei-Wei Tu, Quanming Yao, Huan Zhao, and Isabelle Guyon

Supplemental Experimental Procedures

ChaSuite family and comparison between competition/benchmark

Codabench is the last born of a suite of tools from the open-source "ChaSuite" project[1] (Figure S1), which all have public instances available for free use of charge. "ChaSuite" provides a comprehensive suite of tools for competition and benchmark organizers. *Codabench* is inspired by *CodaLab Competitions*, an open-source platform for running data science competitions, which has been used in hundreds of challenges associated to physics, machine learning, computer vision, natural language processing, health and life sciences, among many other fields. Data science competitions have played an important role for solving machine learning problems both in theory and application (e.g. ImageNet challenge[2], the Netflix Prize[1], etc). Benchmarks can be viewed as a never-ending competition enabling continuous evaluation of methods under the same settings (see Table S1 for a comparison between benchmark and competitions).

Compared with *CodaLab Competitions*, *Codabench* has made significant improvements to better address the organization of benchmarks. The full code has been completely rewritten and the code base is much cleaner and maintainable. We introduce a new "task" concept (as mentioned in Sec 2 and Sec 3) for flexibility and portability purposes. We now support data submission in addition to results and code submission, which makes *Codabench* an important platform for Data Centric AI, which is a new trending paradigm focusing more on the underlying data used to train and evaluate models. We also provide low level APIs to facilitate third party's customization. A new fact sheet system has been added to allow submit more information in an integrated way and the leaderboard now supports multiple modes of display and advanced ranking.

Backward compatibility with previous Codalab. While *Codabench*'s novelty is the feasibility of creating benchmarks, it is fully compatible with previous *CodaLab Competitions*. Competition bundles in the old format e.g. dumped from the Codalab public instance can be re-uploaded to *Codabench*. Competition features such as having multiple-phases (not usually relevance for benchmarks) are supported for compatibility reasons in *Codabench*. Multi-phase challenges help organizers keep participants engaged over long periods of time.

Table S1: **Comparisons of competition and benchmark.** Each row compares both from one perspective.

	Competition	Benchmark
Purpose	Crowdsourcing problems in a short time and harvesting solutions	Continuous fair evaluation, over a long time period, in a unified framework
Phases	Multiple phases	Single phase
Time period	Usually limited	Often never ending
Cooperation & information sharing	Limited due to the competitive nature	As extensive as possible
Submissions	Usually algorithm predictions or algorithm code	Algorithm code or datasets; code or dataset name, description, documentation meta-data and/or fact-sheets; scoring programs for custom analyses
Outcome	Leaderboard with usually a single global ranking based on one score from each team (last or best)	Table with all the submissions made; sorting with multiple scores possible; multiple analyses, graphs, figures, code sharing

Codabench usage: getting started

Using *Codabench* as a participant is straightforward. First, create an account and login on *Codabench*. Then choose an existing benchmark to join following the instructions provided by the organizers. To organize a benchmark, a user can either use the *Codabench* editor or upload a benchmark bundle which is a zip file containing code, dataset, and configuration file. Detailed instructions are found on *Codabench* Documentation. For advanced users who wish to deploy a private instance of *Codabench* please refer to *Codabench* deployment instructions in the same wiki. To illustrate better the benchmark bundle, we provide a simplified bundle example in the next section, which contains ingestion program, scoring program, data, text descriptions and a configuration YAML file. YAML (Yet Another Markup Language) is an extensible markup language that provides convenient syntax for configuration files. See an YAML example in Data S1.

[1] `https://github.com/codalab/codalab-competitions/wiki/Understanding-the-Codalab-Architecture`

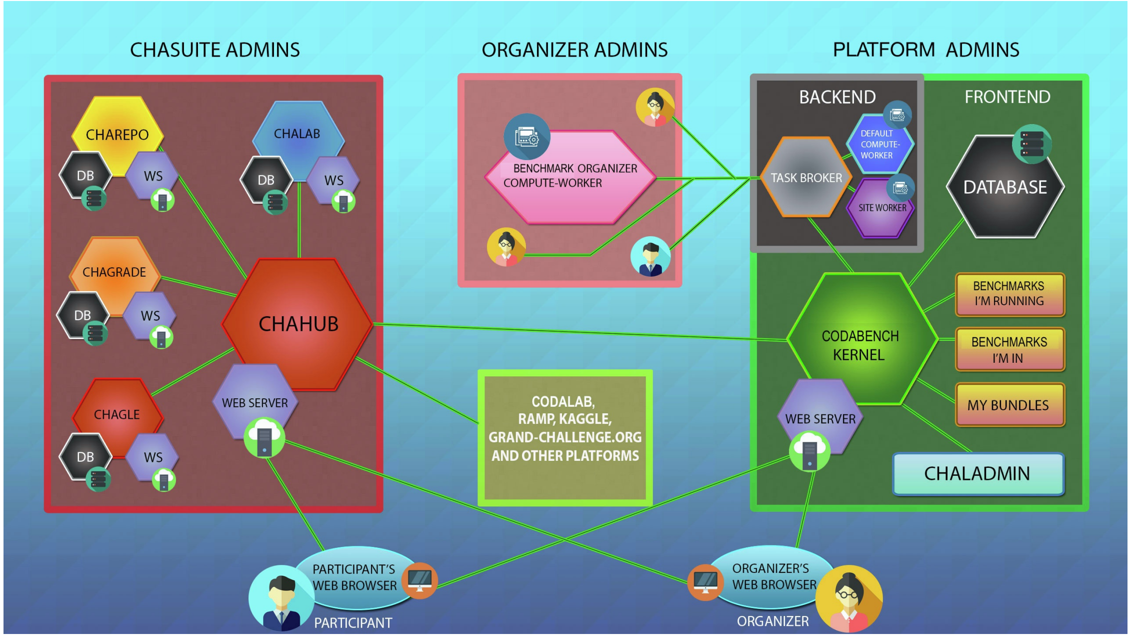

Figure S1: **ChaSuite architecture.** *Codabench* is part of the *CodaLab Competitions* project, including a suite of tools to organize challenges and benchmarks called the“ChaSuite". Right: The kernel of Codabench is interfaced with a web browsers, a database, and a backend dispatching jobs to compute-workers, configured and administered by organizers. Left: The ChaSuite includes an index of competitions and benchmarks (ChaHub) with a search engine (Chagle), a wizard to design challenges (ChaLab), a data repository (ChaRepo), a tool to administer classes (ChaGrade).

Details in a YAML configuration file

Data S1: **Details in a YAML file.** The explanation comment is added at the end of each line in greee.


```yaml
# Sample YAML file based on AutoGraph benchmark
title: 'AutoGraph Benchmark'
description: 'Automated Graph Machine Learning Challenge'
docker_image: nehzux/kddcup2020:v2 # Docker Hub ID
pages:                    # These are "free style" documentation pages
    - title: help         # You can have any title and file name
        file: 'help.html' # You may use HTML or Markdown (.md files)
    - title: overview     # These pages will show up in the benchmark site
        file: 'overview.html'
phases:                   # Benchmarks usually have s single phase
                          # (competitions may have several)
    - index: 0            # Phase order number
        name: 'AutoGraph'
        start: 2021-01-01
        end:   2022-12-31
        tasks:            # Tasks included in this phase
            - 0           # Reference number in task list below,
            - 1           # or absolute reference in Codabench database
        max_submissions: 1000
        max_submissions_per_day: 100
        execution_time_limit_ms: 60000
tasks:                    # Tasks for the above defined phase
    - index: 0
        name: 'Task a'    # For public display on leaderboad
        description: 'Dataset a'    # Private comments
        # Ingestion module:
        ingestion_program: ingestion_program.zip
        input_data: input_data_a.zip
        # Scoring module
        scoring_program: scoring_program.zip
        reference_data: reference_data_a.zip
        # whether the ingestion program is run first, then the
        # scoring program, or the are run in parallel
        ingestion_only_during_scoring: True
    - index: 1
        name: 'Task b'
        description: 'Dataset b'
        # Ingestion module:
        ingestion_program: ingestion_program.zip
        input_data: input_data_b.zip
        # Scoring module
        reference_data: reference_data_b.zip
        scoring_program: scoring_program.zip
        ingestion_only_during_scoring: True
leaderboards:               # Leader board form
    - title: Results        # single leaderboard supported in this version
        key: main           # main key, leave untouched
        columns:
        - title: 'Acc'      # Name of the column displayed
            key: acc        # Data key name used by scoring program
            index: 0        # Order of columns
            sorting: desc   # Sort in descending order
        - title: 'BalAcc'
            key: bacc
            index: 1
            sorting: desc
```

References

[1] Robert M Bell and Yehuda Koren. Lessons from the netflix prize challenge. *Acm Sigkdd Explorations Newsletter*, 9(2):75–79, 2007.

[2] Olga Russakovsky, Jia Deng, Hao Su, Jonathan Krause, Sanjeev Satheesh, Sean Ma, Zhiheng Huang, Andrej Karpathy, Aditya Khosla, Michael Bernstein, Alexander C. Berg, and Fei-Fei Li. Imagenet large scale visual recognition challenge. *International Journal of Computer Vision*, 115(3):211–252, 2015.
