# Supplementary material for: Codabench: Flexible, easy-to-use, and reproducible meta-benchmark platform
Source: Patterns (N Y). 2022 Jun 24;3(7):100543. doi: 10.1016/j.patter.2022.100543 (PMC9278500; doi:10.1016/j.patter.2022.100543)
Supplement: Document S2. Article plus supplemental information [file mmc2.pdf]

Descriptor

Patterns

Codabench: Flexible, easy-to-use, and reproducible meta-benchmark platform

Highlights

- Codabench facilitates flexible, easy, and reproducible benchmarking
- Organizers can customize benchmark design and submission format
- Organizers may host their own platform instance or use the public instance
- Four use cases in diverse domains are introduced to demonstrate the key features

Authors

Zhen Xu, Sergio Escalera, Adrien Pavão, ..., Quanming Yao, Huan Zhao, Isabelle Guyon

Correspondence

xuzhen@4paradigm.com (Z.X.), guyon@chalearn.org (I.G.)

In brief

Fair and flexible benchmarking is a common issue in data-science communities. We develop the Codabench platform for flexible, easy, and reproducible benchmarking. It is open sourced and community driven. With Codabench, we are able to fairly and easily compare algorithms as well as datasets under diverse protocols. The reproducibility is also guaranteed.

Xu et al., 2022, Patterns *3*, 100543
July 8, 2022 © 2022 The Authors.
https://doi.org/10.1016/j.patter.2022.100543

CellPress

Patterns

CellPress
OPEN ACCESS

Descriptor

Codabench: Flexible, easy-to-use, and reproducible meta-benchmark platform

Zhen Xu,[1,7,*] Sergio Escalera,[2] Adrien Pavão,[3] Magali Richard,[4] Wei-Wei Tu,[1] Quanming Yao,[5] Huan Zhao,[1] and Isabelle Guyon[3,6,*]
[1]4Paradigm, Beijing 100085, China
[2]Computer Vision Center, Universitat de Barcelona, 08007 Barcelona, Spain
[3]LISN/CNRS/INRIA, University Paris-Saclay, 91190 Gif-sur-Yvette, France
[4]University Grenoble Alpes, CNRS, UMR 5525, VetAgro Sup, Grenoble INP, TIMC, 38000 Grenoble, France
[5]Tsinghua University, Beijing 100084, China
[6]ChaLearn, Berkeley, CA, USA
[7]Lead contact
*Correspondence: xuzhen@4paradigm.com (Z.X.), guyon@chalearn.org (I.G.)
https://doi.org/10.1016/j.patter.2022.100543

THE BIGGER PICTURE In almost all communities working on data science, researchers face increasingly severe issues of reproducibility and fair comparison. Researchers work on their own version of hardware/software environment, code, and data, and consequently, the published results are hardly comparable. We introduce Codabench, a meta-benchmark platform, that is capable of flexible and easy benchmarking and supports reproducibility. Codabench is an important step toward benchmarking and reproducible research. It has been used in various communities including graph machine learning, cancer heterogeneity, clinical diagnosis, and reinforcement learning. Codabench is ready to help trendy research, e.g., artificial intelligence (AI) for science and data-centric AI.

1 2 3 4 5 **Development/Pre-production:** Data science output has been rolled out/validated across multiple domains/problems

SUMMARY

Obtaining a standardized benchmark of computational methods is a major issue in data-science communities. Dedicated frameworks enabling fair benchmarking in a unified environment are yet to be developed. Here, we introduce Codabench, a meta-benchmark platform that is open sourced and community driven for benchmarking algorithms or software agents versus datasets or tasks. A public instance of Codabench is open to everyone free of charge and allows benchmark organizers to fairly compare submissions under the same setting (software, hardware, data, algorithms), with custom protocols and data formats. Codabench has unique features facilitating easy organization of flexible and reproducible benchmarks, such as the possibility of reusing templates of benchmarks and supplying compute resources on demand. Codabench has been used internally and externally on various applications, receiving more than 130 users and 2,500 submissions. As illustrative use cases, we introduce four diverse benchmarks covering graph machine learning, cancer heterogeneity, clinical diagnosis, and reinforcement learning.

INTRODUCTION

The methodology of unbiased algorithm evaluation is crucial for machine learning and has recently received renewed attention in all data-science scientific communities. Often, researchers have difficulties understanding which dataset to choose for a fair evaluation, with which metrics, under which software/hardware configurations, and on which platforms. The concept of a benchmark itself is not well standardized and includes many settings. For instance, the following may be referred to as a benchmark: a set of datasets, a set of artificial tasks, a set of algorithms, one or several dataset(s) coupled with reference baseline algorithms, a package for fast prototyping algorithms for a specific task, or a hub for compilation of related algorithm implementations. In addition, many benchmarks often integrate new progress by manual verification instead of automatic submission

This is an open access article under the CC BY license (http://creativecommons.org/licenses/by/4.0/).

Table 1. Comparison of various reproducible science platforms

	Flexibility			Easy to use				
Platform	Bundle	Result/code submit	Dataset submit	Easy creation	Open source/ free	API access	Compute queue	Reproducibility
Kaggle	✗	✓	✗	✓	✗	✓	✓	✓
Tianchi	✗	✓	✗	✓	✗	✗	✓	✓
CodaLab	✓	✓	✗	✓	✓	✗	✓	✓
UCI	✗	✗	✓	✗	✓	✗	✗	✓
OpenML	✗	✓	✓	✓	✓	✓	✗	✓
PapersWithCode	✗	✓	✗	✓	✓	✗	✗	✓
DAWNBench	✗	✓	✗	✗	✓	✗	✗	✓
Codabench	✓	✓	✓	✓	✓	✓	✓	✓

Different features are introduced in the section key features of Codabench. Bundle means whether a wrap up is provided for a benchmark such that we could reuse or share. Result/code/dataset submit means whether different submissions are supported to enable flexible tasks. Compute queue means where public or private computation resources could be provided or linked for convenient deployment.

and execution, which delays the benchmark update and requires extra human efforts.

Typical examples of existing frameworks addressing such needs are inventoried in Table 1, including competition platforms, repository hubs, and domain-specific benchmarks. Firstly, competition platforms focus on the participants and provide limited support for organizing general tasks. Famous platforms like Kaggle (https://www.kaggle.com/), Tianchi (https://tianchi.aliyun.com/), and CodaLab (https://codalab.lisn.upsaclay.fr/) organize many data-science challenges, attracting a large number of participants. However, the platform providers retain some control: the organizers do not have full flexibility and control over their competitions. Thus, the experience for organizers is not enjoyable. A comparison between competitions and benchmarks is given in Table S1. Secondly, repository hubs such as UCI repository (https://archive.ics.uci.edu/ml), OpenML,[1] and PapersWithCode (https://paperswithcode.com/) also play an important role for benchmarks and research. They collect large numbers of datasets, methods, and results from academic papers, but reproducibility by running code in given containers (or similar ways) is not guaranteed. Besides the above-mentioned platforms, many domain-specific benchmarks exist, e.g., DAWNBench[2] and KITTI Benchmark Suite.[3] These benchmarks usually focus on a couple of closely related tasks but are not designed to host general benchmarks. In addition, they require repetitive efforts to develop and maintain, which is not always affordable by data-science teams. Thus, to facilitate benchmarking, we need a platform to allow users to flexibly and easily create benchmarks with custom evaluation protocols and custom data formats, with execution in a controlled, reproducible environment, that is totally free and open sourced.

To answer these unmet needs, we developed Codabench, a meta-benchmark platform (Figure 1). A meta-benchmark platform is designed to support general-purpose benchmarks and to facilitate the organization and usage of benchmarks. Codabench takes into account three types of contributors: benchmark participants, benchmark organizers, and platform developers. Benchmark participants submit to different benchmarks, which are prepared and owned by different benchmark organizers. Reproducibility is required at this stage for fair benchmarking. Platform developers contribute different features to Codabench to support diverse benchmarks instead of one specific benchmark, i.e., Codabench is at the meta level of benchmarks. Flexibility and easiness to organize and use benchmarks are thus required at this stage. Codabench realizes these features by implementing an ingestion/scoring programming paradigm, supporting multiple benchmark creation methods and application programming interface (API) access, and using Docker to guarantee reproducibility. Codabench has received over 130 users and 2,500 submissions on 100 tasks including automated machine learning (AutoML),[4] graph machine learning,[5] reinforcement learning (RL),[6] detecting cancer heterogeneity, and training clinicians (https://cancer-heterogeneity.github.io/). Multiple illustrative use cases are introduced. Codabench is an important step toward reproducible research and should meet the interest of all areas of data science.

Method: Design of Codabench

Codabench is a meta-benchmark platform that provides a flexible, easy-to-use, and reproducible benchmarking service that is publicly and freely available for everyone. In Codabench, benchmarks are implemented by benchmark bundles, which contain one or several tasks. The concept of a task is newly introduced, which is the minimal unit for composing a benchmark (bundle). A task consists of an ‘‘ingestion module’’ (including an ingestion program and input data), a ‘‘scoring module’’ (including a scoring program and reference data, invisible to the participant's submission), a baseline solution with sample data, and meta-data information if needed. Tasks in Codabench may be programmed in any programming language in any custom way and are run in a docker specified by organizers. Figure 2 provides a detailed description of Codabench internal interaction logistics.

Take supervised-learning tasks as an example. A typical usage is that benchmark participants submit a class (e.g., a Python object) ‘‘z’’, with 2 methods, z.fit and z.predict, similarly to scikit-learn objects.[7] The ingestion program reads data, calls z.fit with labeled training data and z.predict with unlabeled test data (labeled training data and unlabeled test data being part of the so-called ‘‘input data’’), then outputs predictions. The scoring program reads the predictions and

CellPress
OPEN ACCESS

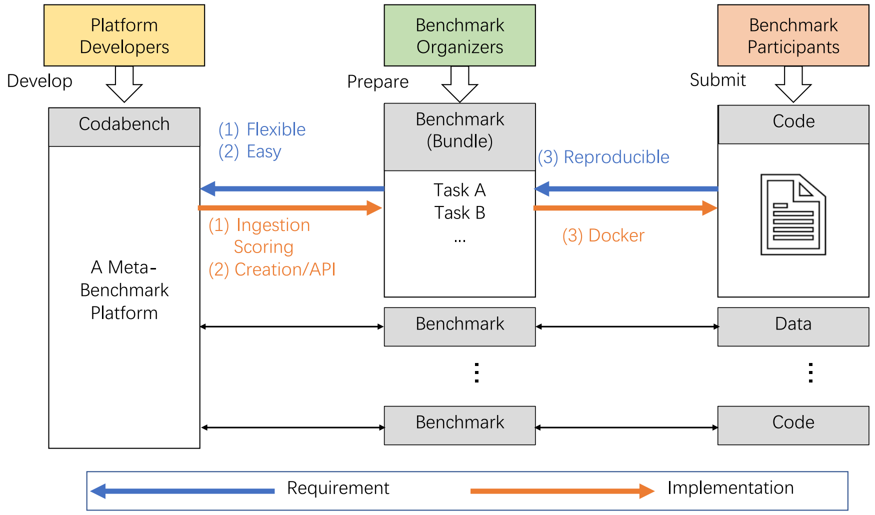

Figure 1. Overview of Codabench
A meta-benchmark platform has three types of contributors: platform developers (in yellow), benchmark organizers (in green), and benchmark participants (in red). Codabench is at the meta level to support diverse benchmarks. Each benchmark is implemented by a benchmark bundle that contains one or more tasks.

evaluates them based on custom scoring metric(s) using the test labels (called "reference data"). Other application usages are possible, including transposed benchmarks, where datasets are submitted by participants instead of algorithms, while the organizers supply a set of algorithms, and RL benchmarks, where the ingestion program plays the role of an agent wrapping around the submission of the participant and interacting with a world (scoring program) in a specific way.

The reader is referred to the Codabench official repository (https://github.com/codalab/codabench/), where the code and complete documentation are found. In the supplemental information, we also include instructions and references to get started. To use the public instance of Codabench, please visit the Codabench website. To test and install locally, the instructions are given in the README file of the official repository. The Codabench code is released under an Apache 2.0 license.

RESULTS

Key features of Codabench

Codabench is task oriented. Using tasks, the organizers have the flexibility of implementing any benchmark protocol, with any dataset format and API, or even using data-generating models, allowing them to organize RL challenges. In this section, we introduce the key features of Codabench contributing to its flexibility, ease, and reproducibility, as shown in Figure 1. Codabench also supports custom leaderboards and has full documentation of usage.

Flexibility

Codabench supports flexible benchmark types including results submission, code submission, and even dataset submission. Benchmarks on Codabench are organized by bundles containing all the information of a benchmark.

Bundle (hosting a benchmark): a benchmark bundle is a zip file containing all necessary constituents of a benchmark, including tasks, documentation, and configuration settings (such as leaderboard settings). A Codabench bundle may include a single or multiple tasks. A classical benchmark is usually single task while AutoML,[4] transfer learning,[8] and meta learning[9] benchmarks are multitask.

Results or code submission: "classic" Codabench benchmarks are either with result or code submission. On one hand, result submissions are used when organizers wish that participants use their own computational resources. In supervised-learning competitions, participants would supply, e.g., predictions of output values on some test datasets. Other types of results may be supplied, for instance, high-resolution images in a hyper-resolution challenge for which inputs are low-resolution images. On the other hand, if the organizers wish to run all algorithms in a uniform manner on the platform, Codabench allows the participants to make code submissions. The submitted software is run in a docker supplied by the organizers, either on the default compute worker or on compute workers supplied by the organizers. This code-submission design allows organizers to provide suitable computational resources (e.g., GPUs) and improve reproducibility.

Dataset submission: the role of the dataset and algorithm can be transposed with Codabench to facilitate data-centric artificial intelligence (AI) (https://datacentricai.org/), which is a trending research topic that cares about the quality and usage of data. In a classic benchmark, organizers provide datasets, and participants submit algorithms. In a transposed benchmark, participants submit datasets, and organizers provide reference algorithms. A classic benchmark will have a leaderboard with datasets in columns that grows by adding more lines as algorithm submissions are made. In a transposed dataset-submission benchmark, the leaderboard will have algorithms in columns, and lines are added as more datasets are submitted. With Codabench's transposed benchmark, it is easier to try different data-augmentation and -processing methods with fixed algorithms as test cases.

Easy to use

To facilitate an easiness of using the system, we provided several tools to help the benchmark organizers create a benchmark. A platform editor is provided to develop a benchmark, which provides simple user interfaces to prepare data, code, and other configurations. As a second option, the user can upload a locally prepared benchmark bundle to facilitate local debugging and testing. Once uploaded, a benchmark can further be modified using the platform editor. An existing benchmark can be saved as another bundle, which facilitates sharing and portability. Similar benchmark bundles can be easily prepared with shared template bundles. Codabench is open sourced and free to use.

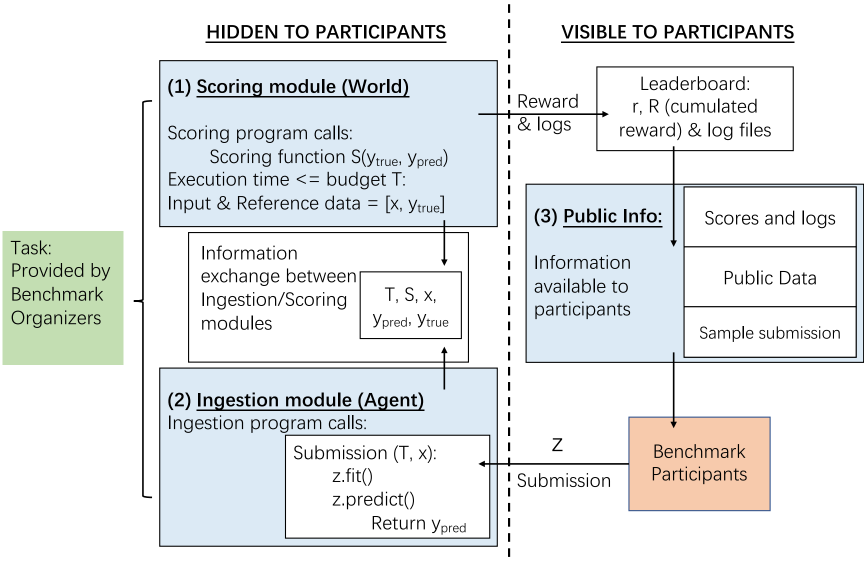

Figure 2. Operational Codabench workflow
Left: task module specified by the benchmark organizers, executed on the platform. Right: web interface with participants permitted to make submissions and retrieve results. Numerated blocks are specified by the benchmark organizers. They include (1) a scoring module, (2) an ingestion module, (3) and public information. An intermediate block also exists for information exchange of time budget, scoring, input data, ground-truth data, and predictions. Red bottom-right block: participant prepares a submission "z" uploaded to the platform. The submission is then executed by the ingestion program. The role of the scoring program is to produce scores that are then displayed on the leaderboard.

APIs to external clients: we provide APIs for interacting with the platform, including "robot" submissions via command lines, without going through the regular Codabench web interface, and this is likewise a programmatic way of recuperating results directly without going through the leaderboard.

Dedicated computing queues: the public instance of Codabench provides default compute workers. Organizers can also create a dedicated job queue and connect it to their own CPU or GPU compute workers.

Reproducibility

Codabench makes extensive use of dockers (https://www.docker.com/) to guarantee reproducibility. Benchmark organizers specify the docker image by providing its docker hub name and tag. Docker wraps all the software dependencies into a lightweight virtual image. Once a docker is provided by the benchmark organizer, the program can be run inside a docker that contains exactly the same installed packages. This docker will be pulled every time a benchmark's program is executed. Different benchmarks could use different dockers, which are usually provided by organizers. We also provide a default docker for more general benchmarks' usage or people who are not familiar with dockers.

Other features

Custom leaderboard: to better facilitate benchmarks, the leaderboard is fully customizable and can handle multiple datasets and multiple custom scoring functions. We provide multiple ways to display submissions (best per participant, multiple submissions per participant, etc.), and the leaderboard can flexibly rank submissions by average score, per task, per submetric of a certain task, etc.

Documentation: the documentation (https://github.com/codalab/codabench/wiki) provides detailed help for different types of contributors. For benchmark participants, we provide instructions to join and submit to a benchmark. For benchmark organizers, we provide annotated instructions for organizing benchmarks. Several benchmark-bundle templates, from simple to advanced, are also available to ease the technical aspects of building a benchmark and to let people concentrate on scientific aspects of the benchmark. For platform developers, we explain more technical specifications on technology stack and provide ways to integrate to the project. Platform developers are contacted via the GitHub issues and pull requests to solve issues encountered in daily usage.

Use cases of Codabench

Codabench has been used not only internally at 4Paradigm and LISN Lab for tasks of AutoML,[4] graph machine learning,[5] RL,[6] speech recognition,[10] and weakly supervised learning[11] but also externally by University Grenoble Alphes for hosting scientific benchmarks in cancer heterogeneity and training clinicians. Codabench has received more than 130 users and 2,500 submissions distributing on various applications. In this section, we introduce 4 use cases of Codabench, aiming at demonstrating different Codabench features and capabilities. A visual illustration is given in Figure 3.

Use case 1: AutoGraph benchmark

In this section, we introduce automated graph machine learning (AutoGraph) benchmark, which targets automated node classification methods on diverse dataset scenarios. With this use case, we show a set of fundamental features of Codabench: (1) the code submission mode, (2) reproducibility guaranteed by docker, (3) flexible benchmark-bundle configuration with multiple tasks, and (4) customizable computational resources.

Background: graph machine learning has been a very hot topic due to the ubiquity of graph-structured data, e.g., social networks,[12] molecule graphs,[13] knowledge graphs,[14] etc. Typical tasks of graph data include node level (node classification), edge level (link prediction), and graph level (graph regression/classification). The task of our benchmark here is node classification, i.e., given a graph where some nodes are labeled and the rest are unlabeled, we want to predict the classes of the unlabeled nodes. In addition, we require the algorithm to perform well on a set of datasets instead of just one dataset. This leads to automated-graph-machine-learning problem, which we call AutoGraph.

Implementation: the AutoGraph benchmark is a typical code-submission use case. It focuses on AutoML methods,[4] which requires more than one dataset to be evaluated together. A Codabench bundle is, by design, flexible with multiple tasks, each of

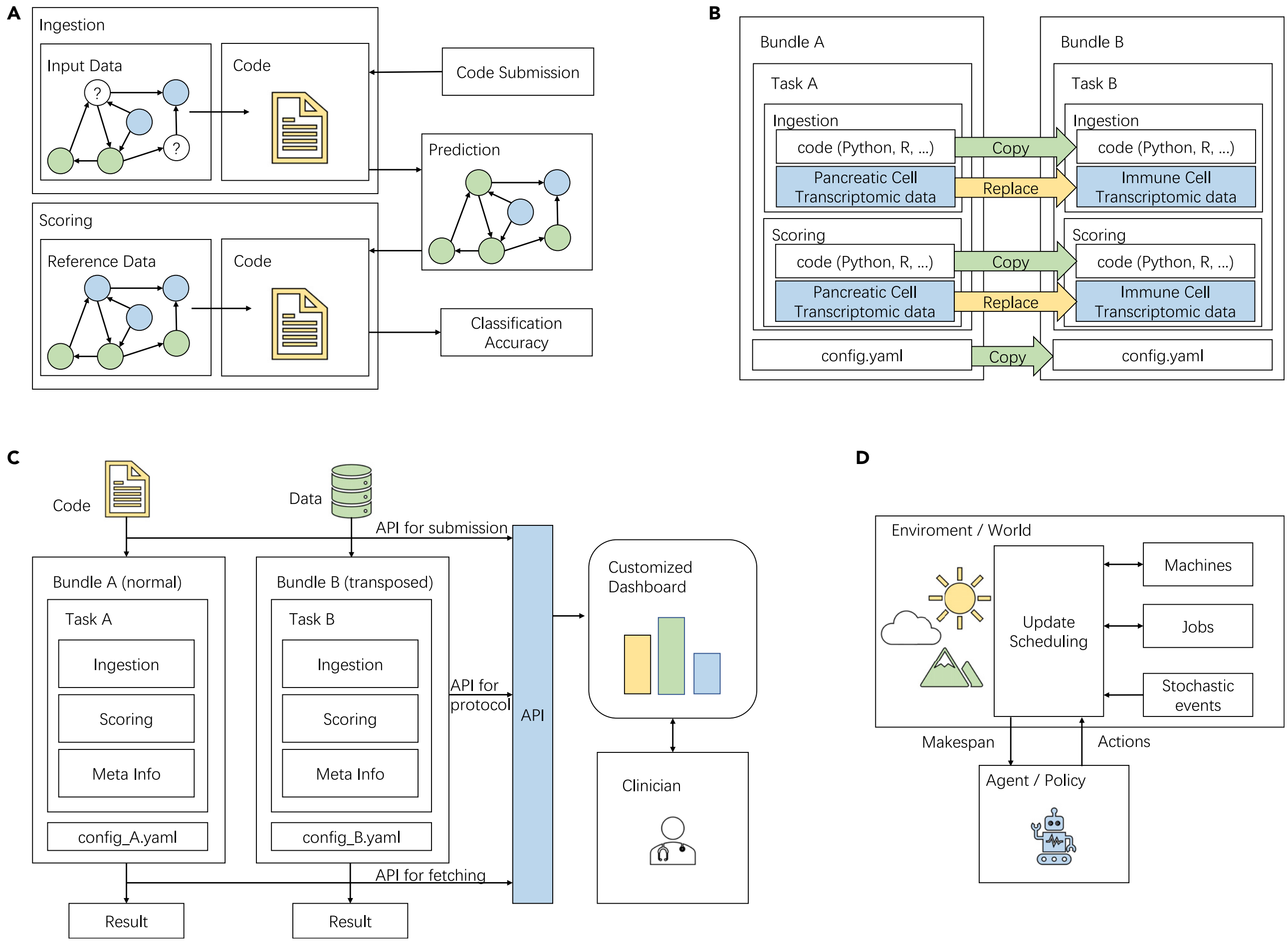

Figure 3. Use-case illustrations

Four use cases are introduced: (A) AutoGraph, (B) DECONbench, (C) COMETH, and (D) job scheduling. The use-case details are introduced in the section Use cases of Codabench.

which contains a separate dataset. We also provide a docker hosted on DockerHub, which could be pulled automatically by the Codabench platform to run each algorithm submission and be used for researchers' local development. Every time a new method is uploaded, a new docker container instance will be called to independently run the method for each dataset. In this way, we make sure every algorithm is fairly run under the same setting and that the whole process can be fully reproduced on other machines. Codabench is designed to adapt to any docker-enabled computational resource (local machine, cluster server, cloud machines, etc.). We currently host the AutoGraph benchmark on Codabench with free computational resources thanks to Google's sponsorship, encouraging everyone to contribute. Besides, the datasets are also available to the public for local usage and further benchmarking on GitHub (https://github.com/AutoML-Research/AutoGraph-KDDCup2020) and Kaggle. We uploaded the solutions of the winners of the challenge as baselines. Since the benchmark datasets are already released, users can also run complementary experiments on their local computers and debug mode easily, thus making progress more rapidly. With the AutoGraph benchmark, we provide researchers and practitioners the possibility to showcase results in a public venue.

Use case 2: DECONbench benchmark

In this section, we introduce the DECONbench benchmark.[15] DECONbench aims at benchmarking algorithms inferring tumor cellular composition from molecular data. Here, we highlight two features of Codabench: (1) flexibility of benchmark bundles (in this use case, another task and programming language R supported) and (2) reusability and portability of benchmark bundles.

Background: successful treatment of cancer is still a challenge, and this is partly due to a wide heterogeneity of tumor cellular compositions across patient population. Tumors are made up of cells with different identities and origins. Cancer cells evolving in a dynamic environment consist of aberrant non-cancerous cells, such as blood vessels or immune cells. Tumor cellular composition is difficult to observe and quantify, as it is hidden inside the bulk molecular profiles of the samples, with millions of cells present in the tumor (and not only cancer cells) contributing to the bulk recorded signals. Taking advantage of the large amount of molecular data publicly available, a wide number of supervised and unsupervised algorithms have been recently developed to estimate tumor cellular composition.[16–18] DECONbench is a series of benchmarks dedicated to the quantification of tumor composition, focusing on estimating cell types and proportion in biological

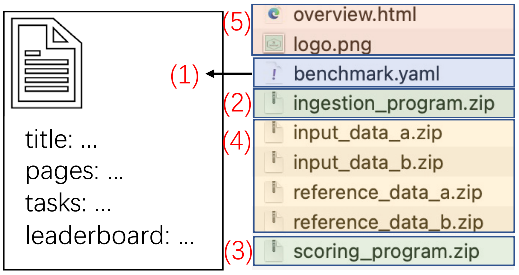

Figure 4. Bundle structure
The details of benchmark.yaml is given in Data S1.

samples using multimodal molecular data. Participants have to identify an estimation of the tumor composition, i.e., a matrix of the estimated proportion of each deconvoluted cell type (rows) for each sample (columns). The discriminating metric is the mean absolute error (MAE) between prediction and ground-truth matrix. Note that the DECONbench series is optimized to run methods developed in the statistical programming language R.

Implementation: using the Codabench platform, the COMETH consortium firstly developed a benchmark for continuous evaluation of computational methods based on epigenomic data (https://www.codabench.org/competitions/174). Since we are at the same time interested in other modalities of data under similar tasks, it would be ideal to reuse previously created bundles instead of going through everything again. Thanks to the portability of the Codabench bundle design, we only need to replace the data files and adjust slightly the protocol code. All other configuration files can be reused. As a result, this first benchmark was easily cloned and extended to similar benchmarks using other types of data, e.g., all-cell-type transcriptomic data (https://www.codabench.org/competitions/147), immune-cell-type transcriptomic data (https://www.codabench.org/competitions/148), and all-cell-type multimodal transcriptomic and epigenomic data (https://www.codabench.org/competitions/237).

Use case 3: COMETH benchmark

In this section, we introduce the COMETH benchmark, which is motivated by real clinical application and is an exciting step toward data-centric AI. With this use case, we show that (1) Codabench supports a transposed benchmark consolidating data-centric AI and (2) the provided API interaction opens a window for other customization scenarios.

Background: when it comes to clinical application, it is often necessary for health-data scientists and clinicians to identify the most suitable existing method to be applied on a given dataset. In this case, we focus more on the data instead of algorithmic development, which aligns with data-centric AI. Usually, the clinicians do not (need to) know much about the algorithm details. Instead, they have access to newly available data and want to apply the most relevant algorithms on their new data. There is thus a need to provide an effective tool displaying the evaluation of state-of-the-art algorithms on reference datasets and enabling their application on new datasets. This will guide and facilitate the appropriate use of these algorithms by non-expert clinicians. Note that these algorithms are usually provided by benchmark organizers who are domain experts on certain tasks.

Implementation: to solve this question, the COMETH consortium developed the COMETH benchmark (https://www.codabench.org/competitions/218), a transposed challenge in which datasets should be submitted to be evaluated against existing algorithms (i.e., tasks in the Codabench design). For instance, the COMETH benchmark provides a series of recent deconvolution algorithms that are able to quantify tumor heterogeneity.[16–18] Clinicians aiming to quantify tumor heterogeneity from molecular data can submit their dataset of interest to the COMETH benchmark and retrieve the corresponding outputs in a fully reproducible environment. To facilitate the use of this functionality by clinicians who are less familiar with data-science programming details, the COMETH benchmark has been connected to an external client displaying a user-friendly web dashboard. The external client is able to send requests to users directly on the COMETH benchmark using APIs provided by Codabench and return the generated results from all reference algorithms. This feature strongly contributes to a direct transfer of knowledge between data scientists and healthcare professionals. This design was used at a winter school for training clinicians and data scientists (https://cancer-heterogeneity.github.io/cometh.html).

Use case 4: Job scheduling benchmark

We lastly introduce another use case, the job scheduling benchmark, which focuses on RL and operational research. With this use case, we show that Codabench is RL friendly with the help of flexible designs of benchmark bundles.

Background: we consider the problem of dynamic job shop scheduling.[19–21] The task is to allocate a set of jobs to a set of machines to achieve the shortest execution time, i.e., makespan. Each job has a pre-determined operation sequence to be executed on certain machines. To mimic real-life scenarios, we add stochastic machine-down events to the problem. This task is usually formulated as a sequential decision-making problem and fits easily to RL. We thus expect an agent making decisions on how to better schedule the jobs in minimal time. The reward depends on the makespan.

Implementation: as explained in the section method: design of Codabench, our design of bundles and an ingestion/scoring program makes it very natural and flexible for RL problems. We easily use the scoring program as an environment that evaluates a job schedule and returns a makespan as reward. The ingestion program serves as an agent and makes decisions on job scheduling based on the received reward.

A concrete benchmark-bundle example

In this section, we provide a concrete benchmark-bundle example to show how simple it is to organize benchmarks on Codabench. A bundle consists of five parts, as in Figure 4: (1) a YAML configuration file (https://yaml.org/), (2) an ingestion program, (3) a scoring program, (4) data, and (5) additional files for description.

The ingestion program usually reads data and a participant's submission. It calls the participant's method on the dataset and produces predictions to a shared space. The scoring program usually reads the ingestion program's output and evaluates with respect to (w.r.t.) ground truth according to an organizer's customized metric. It finally writes scores to a text file, which will be read by the platform and be displayed on a leaderboard.

The data contain input data (in supervised learning, they are usually X_train, y_train, and X_test) and reference data (in supervised learning, it is usually y_test). Both are zipped into separate files. The additional files are just text or figure files for organizers to provide other information, e.g., instructions, references, logo, etc. A final YAML file connects all previous parts and provides more configurations for the benchmark. A simplified YAML file is given in Data S1. It contains general configurations like title, logo image, docker image, which HTMLs are to be displayed, leaderboard configuration (e.g., which metrics will be used in the leaderboard), and tasks. Each task is by itself a complete unit for running. It contains name, ID, ingestion program, scoring program, input data, reference data.

DISCUSSION

Codabench is a new meta-benchmark platform for data-science communities. Codabench is compatible with diverse tasks (including supervised learning and RL) and supports result, code, and dataset submission. It is easy to use Codabench, and reproducibility is guaranteed by dockers. Codabench has a public instance free for use, deployed at Université Paris-Saclay, but can also be deployed locally with the technology stack provided in documentation. Hosting, maintaining, and further developing the platform is funded by grants and donations. As real-world scenarios, we introduce 4 benchmark use cases illustrating the flexibility, ease of use, reproducibility, and other features of Codabench. We also note that tremendous other tasks could be integrated into Codabench as well including electroencephalogram (EEG) classification, drug discovery and property prediction, and dynamic simulation for weather, traffic, fluid, etc., which are important tasks toward AI for science.

The current limitations of Codabench are mainly as follows. First, since it is relatively new, we do not have yet an active community of organizers and benchmark participants. We need more users' feedback to polish up our user interface and documentation. Second, although supported by design, we have not yet had a distributed computation scenario where complex multi-node compute workers are used. This could enrich our benchmark template library with benchmarks for algorithm parallelization. Thirdly, although Codabench supports both code and dataset submissions, we do not currently allow users to extend the leaderboard in both directions simultaneously, i.e., it does not allow users to submit both code and datasets at the same time. This feature could largely increase the user experience of the platform. Lastly, Codabench does not yet support hardware-related benchmarks or human-in-the-loop benchmarks, which could be interesting to consider in the future.

Potentially harmful uses of Codabench could result from poor benchmark designs (e.g., no scientific question is asked by hosting a benchmark) or bad data collections (e.g., data license, data quality), as in any machine-learning project. We are working on an open-access book (to appear in 2022) on best practices for designing challenges and benchmarks including data preparation, task evaluation, etc. and for post-challenge/post-benchmark analysis.

Further works include providing more comprehensive usage templates illustrating features such as (1) splitting an algorithm workflow into submodules and scoring the effectiveness of the modules individually (e.g., with ablation or sensitivity analysis), (2) providing templates of fact sheets to extract information about algorithms (similar to datasheets for datasets but for algorithms), and (3) providing guidelines to benchmark participants to produce enriched detailed results, amenable to meta-analyses.

EXPERIMENTAL PROCEDURES

Resource availability

Lead contact

The lead contact is Zhen Xu (xuzhen@4paradigm.com), who is a research scientist at 4Paradigm, Beijing, China.

Materials availability

This study did not generate new materials.

Data and code availability

The code of Codabench is available at https://github.com/codalab/codabench. This work does not introduce new datasets. For creating benchmarks, organizers should prepare their own datasets.

SUPPLEMENTAL INFORMATION

Supplemental information can be found online at https://doi.org/10.1016/j.patter.2022.100543.

ACKNOWLEDGMENTS

The Codabench project shares the same community governance as CodaLab Competitions. The openness of Codabench is total: an Apache 2.0 license is used, the source code is on GitHub, and the development framework and all the used components are open sourced. Codabench has received important contributions from many people who did not co-author this paper, and we would like to thank their efforts in making Codabench what it is today, including early CodaLab Competitions developers and contributors (listed alphabetically): Pujun Bhatnagar, Justin Carden, Richard Caruana, Francis Cleary, Xiawei Guo, Ivan Judson, Lori Ada Kilty, Shaunak Kishore, Stephen Koo, Percy Liang, Zhengying Liu, Pragnya Maduskar, Simon Mercer, Arthur Pesah, Christophe Poulain, Lukasz Romaszko, Laurent Senta, Lisheng Sun, Sebastien Treguer, Cedric Vachaudez, Evelyne Viegas, Paul Viola, Erick Watson, Tony Yang, Flavio Zhingri, and Michael Zyskowski. We would like to particularly thank the people who contributed to the design, development, and testing of Codabench including (listed alphabetically) Alexis Arnaud, Xavier Baró, Feng Bin, Yuna Blum, Eric Carmichael, Laurent Darré, Hugo Jair Escalante, Sergio Escalera, Eric Frichot, Yuxuan He, James Keith, Anne-Catherine Letournel, Shouxiang Liu, Zhenwu Liu, Adrien Pavao, Magali Richard, Tyler Thomas, Nic Threfts, Bailey Trefts, Catherine Wallez, and Lanning Wei. The Université Paris-Saclay is hosting the main instance of Codabench. Funding and support have been received via several research grants, including Big Data Chair of Excellence FDS Paris-Saclay, Paris Région Ile-de-France, EU EIT projects HADACA and COMETH, United Health Foundation INCITE project, ANR Chair of Artificial Intelligence HUMANIA ANR-19-CHIA-0022, the Spanish project PID2019-105093GB-I00, ICREA under the ICREA Academia program, INSERM Cancer project ACACIA 232717, MIAI @Grenoble Alpes (ANR-19-P3IA-0003), 4Paradigm, ChaLearn, Microsoft, and Google. We also appreciate the following people and institutes for their open-source datasets that are used in our use cases: Andrew McCallum, C. Lee Giles, Ken Lang, Tom Mitchell, William L. Hamilton, Maximilian Mumme, Oleksandr Shchur, David D. Lewis, William Hersh, Just Research and Carnegie Mellon University, NEC Research Institute, Carnegie Mellon University, Stanford University, Technical University of Munich, AT&T Labs, and Oregon Health Sciences University. We are also very grateful to Joaquin Vanschoren for fruitful discussions.

AUTHOR CONTRIBUTIONS

Conceptualization, Z.X., S.E., A.P., and I.G.; methodology, Z.X. and I.G.; validation and investigation, all authors; resources and data curation, Z.X., M.R., W.-W.T., and I.G.; writing – original draft, all authors; writing – review & editing,

Z.X., Q.Y., M.R., and I.G.; visualization, Z.X., Q.Y., and I.G.; supervision and project administration, I.G.; funding acquisition, W.-W.T. and I.G.

DECLARATION OF INTERESTS

Z.X., W.-W.T., and H.Z. are employed by 4Paradigm, China. I.G. is president of ChaLearn, a not-for-profit organization dedicated to running challenges in machine learning. ChaLearn is a tax-exempt not-for-profit organization under section 501(c)(3) of the US IRS code of the United States. It derived no profit from sponsoring this research.

Received: February 25, 2022
Revised: March 21, 2022
Accepted: June 3, 2022
Published: June 24, 2022

REFERENCES

1. Vanschoren, J., van Rijn, J.N., Bischl, B., and Torgo, L. (2014). Openml: networked science in machine learning. SIGKDD Explor. *15*, 49–60. https://doi.org/10.1145/2641190.2641198.
2. Coleman, C., Kang, D., Narayanan, D., Nardi, L., Zhao, T., Zhang, J., Bailis, P., Olukotun, K., Christopher, R., and Zaharia, M. (2019). Analysis of dawnbench, a time-to-accuracy machine learning performance benchmark. ACM SIGOPS Oper. Syst. Rev. *53*, 14–25. https://doi.org/10.1145/3352020.3352024.
3. Geiger, A., Lenz, P., and Urtasun, R. (2012). Are we ready for autonomous driving? The KITTI vision benchmark suite. In IEEE Conference on Computer Vision and Pattern Recognition (IEEE Computer Society), pp. 3354–3361.
4. F. Hutter, L. Kotthoff, and J. Vanschoren, eds. (2019). Automated Machine Learning - Methods, Systems, Challenges (Springer).
5. Hu, W., Fey, M., Zitnik, M., Dong, Y., Ren, H., Liu, B., Catasta, M., and Leskovec, J. (2020). Open graph benchmark: datasets for machine learning on graphs. In Advances in Neural Information Processing Systems.
6. Sutton, R.S., and Barto, A.G. (2018). Reinforcement Learning: An Introduction, Second edition (The MIT Press).
7. Pedregosa, F., Varoquaux, G., Gramfort, A., Michel, V., Thirion, B., Grisel, O., Blondel, M., Prettenhofer, P., Weiss, R., Dubourg, V., et al. (2011). Scikit-learn: machine learning in python. J. Mach. Learn. Res.
8. Pan, S.J., and Yang, Q. (2009). A survey on transfer learning. In IEEE Transactions on Knowledge and Data Engineering (IEEE).
9. Vilalta, R., and Drissi, Y. (2002). A perspective view and survey of meta-learning. Artif. Intell. Rev. *18*, 77–95. https://doi.org/10.1023/a:1019956318069.
10. Povey, D., Ghoshal, A., Boulianne, G., Burget, L., Glembek, O., Goel, N., Hannemann, M., Motlicek, P., Qian, Y., Schwarz, P., et al. (2011). The kaldi speech recognition toolkit. In IEEE workshop on automatic speech recognition and understanding.
11. Zhou, Z.-H. (2018). A Brief Introduction to Weakly Supervised Learning (National Science Review).
12. Hamilton, W.L., Ying, R., and Leskovec, J. (2017). Representation learning on graphs: methods and applications. IEEE Data Eng. Bull. *40*, 52–74.
13. Mansimov, E., Mahmood, O., Kang, S., and Cho, K. (2019). Molecular geometry prediction using a deep generative graph neural network. Sci. Rep. *9*, 1–13.
14. Antoine, B., Usunier, N., Garcia-Duran, A., Weston, J., and Yakhnenko, O. (2013). Translating embeddings for modeling multi-relational data. Adv. Neural Inf. Process. Syst. *26*, 2787–2795.
15. Decamps, C., Arnaud, A., Petitprez, F., Ayadi, M., Baurès, A., Armenoult, L., Escalera, S., Guyon, I., Nicolle, R., Tomasini, R., and de Reyniès, A. (2021). Deconbench: a benchmarking platform dedicated to deconvolution methods for tumor heterogeneity quantification. BMC Bioinf. *22*, 473. https://doi.org/10.1186/s12859-021-04381-4.
16. Avila Cobos, F., Alquicira-Hernandez, J., Powell, J.E., Mestdagh, P., and De Preter, K. (2020). Benchmarking of cell type deconvolution pipelines for transcriptomics data. Nat. Commun. *11*, 1–14.
17. Cantini, L., Kairov, U., de Reyniès, A., Barillot, E., Radvanyi, F., and Zinovyev, A. (2019). Assessing reproducibility of matrix factorization methods in independent transcriptomes. Bioinformatics *35*, 4307–4313. https://doi.org/10.1093/bioinformatics/btz225.
18. Decamps, C., Privé, F., Bacher, R., Jost, D., Waguet, A., Houseman, E.A., Andres Houseman, E., Lurie, E., Lutsik, P., Milosavljevic, A., et al. (2020). Guidelines for cell-type heterogeneity quantification based on a comparative analysis of reference-free dna methylation deconvolution software. BMC Bioinf. *21*, 16. https://doi.org/10.1186/s12859-019-3307-2.
19. Aydin, M.E., and Öztemel, E. (2000). Dynamic job-shop scheduling using reinforcement learning agents. Robot. Autonomous Syst. *33*, 169–178.
20. Jain, A.S., and Meeran, S. (1999). Deterministic job-shop scheduling: past, present and future. Euro. J. Operat. Res. *11*, 390–434.
21. Ramasesh, R. (1990). Dynamic job shop scheduling: a survey of simulation research. Omega *18*, 43–57.

Patterns, Volume *3*

Supplemental information

Codabench: Flexible, easy-to-use,

and reproducible meta-benchmark platform

Zhen Xu, Sergio Escalera, Adrien Pavão, Magali Richard, Wei-Wei Tu, Quanming Yao, Huan Zhao, and Isabelle Guyon

Supplemental Experimental Procedures

ChaSuite family and comparison between competition/benchmark

Codabench is the last born of a suite of tools from the open-source "ChaSuite" project[1] (Figure S1), which all have public instances available for free use of charge. "ChaSuite" provides a comprehensive suite of tools for competition and benchmark organizers. *Codabench* is inspired by *CodaLab Competitions*, an open-source platform for running data science competitions, which has been used in hundreds of challenges associated to physics, machine learning, computer vision, natural language processing, health and life sciences, among many other fields. Data science competitions have played an important role for solving machine learning problems both in theory and application (e.g. ImageNet challenge[2], the Netflix Prize[1], etc). Benchmarks can be viewed as a never-ending competition enabling continuous evaluation of methods under the same settings (see Table S1 for a comparison between benchmark and competitions).

Compared with *CodaLab Competitions*, *Codabench* has made significant improvements to better address the organization of benchmarks. The full code has been completely rewritten and the code base is much cleaner and maintainable. We introduce a new "task" concept (as mentioned in Sec 2 and Sec 3) for flexibility and portability purposes. We now support data submission in addition to results and code submission, which makes *Codabench* an important platform for Data Centric AI, which is a new trending paradigm focusing more on the underlying data used to train and evaluate models. We also provide low level APIs to facilitate third party's customization. A new fact sheet system has been added to allow submit more information in an integrated way and the leaderboard now supports multiple modes of display and advanced ranking.

Backward compatibility with previous Codalab. While *Codabench*'s novelty is the feasibility of creating benchmarks, it is fully compatible with previous *CodaLab Competitions*. Competition bundles in the old format e.g. dumped from the Codalab public instance can be re-uploaded to *Codabench*. Competition features such as having multiple-phases (not usually relevance for benchmarks) are supported for compatibility reasons in *Codabench*. Multi-phase challenges help organizers keep participants engaged over long periods of time.

Table S1: **Comparisons of competition and benchmark.** Each row compares both from one perspective.

	Competition	Benchmark
Purpose	Crowdsourcing problems in a short time and harvesting solutions	Continuous fair evaluation, over a long time period, in a unified framework
Phases	Multiple phases	Single phase
Time period	Usually limited	Often never ending
Cooperation & information sharing	Limited due to the competitive nature	As extensive as possible
Submissions	Usually algorithm predictions or algorithm code	Algorithm code or datasets; code or dataset name, description, documentation metadata and/or fact-sheets; scoring programs for custom analyses
Outcome	Leaderboard with usually a single global ranking based on one score from each team (last or best)	Table with all the submissions made; sorting with multiple scores possible; multiple analyses, graphs, figures, code sharing

Codabench usage: getting started

Using *Codabench* as a participant is straightforward. First, create an account and login on *Codabench*. Then choose an existing benchmark to join following the instructions provided by the organizers. To organize a benchmark, a user can either use the *Codabench* editor or upload a benchmark bundle which is a zip file containing code, dataset, and configuration file. Detailed instructions are found on *Codabench* Documentation. For advanced users who wish to deploy a private instance of *Codabench* please refer to *Codabench* deployment instructions in the same wiki. To illustrate better the benchmark bundle, we provide a simplified bundle example in the next section, which contains ingestion program, scoring program, data, text descriptions and a configuration YAML file. YAML (Yet Another Markup Language) is an extensible markup language that provides convenient syntax for configuration files. See an YAML example in Data S1.

[1] https://github.com/codalab/codalab-competitions/wiki/Understanding-the-Codalab-Architecture

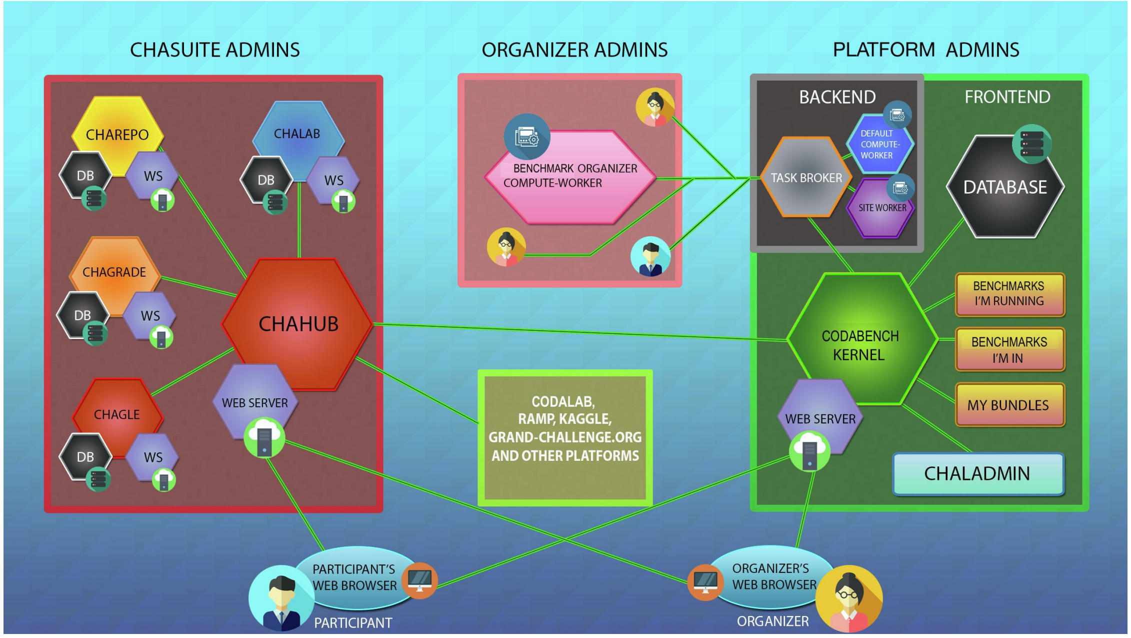

Figure S1: **ChaSuite architecture.** *Codabench* is part of the *CodaLab Competitions* project, including a suite of tools to organize challenges and benchmarks called the“ChaSuite". Right: The kernel of Codabench is interfaced with a web browsers, a database, and a backend dispatching jobs to compute-workers, configured and administered by organizers. Left: The ChaSuite includes an index of competitions and benchmarks (ChaHub) with a search engine (Chagle), a wizard to design challenges (ChaLab), a data repository (ChaRepo), a tool to administer classes (ChaGrade).

Details in a YAML configuration file

Data S1: **Details in a YAML file.** The explanation comment is added at the end of each line in greee.

```yaml
# Sample YAML file based on AutoGraph benchmark
title: 'AutoGraph Benchmark'
description: 'Automated Graph Machine Learning Challenge'
docker_image: nehzux/kddcup2020:v2 # Docker Hub ID
pages:                    # These are "free style" documentation pages
    - title: help         # You can have any title and file name
        file: 'help.html' # You may use HTML or Markdown (.md files)
    - title: overview     # These pages will show up in the benchmark site
        file: 'overview.html'
phases:                   # Benchmarks usually have s single phase
                          # (competitions may have several)
    - index: 0            # Phase order number
        name: 'AutoGraph'
        start: 2021-01-01
        end:   2022-12-31
        tasks:            # Tasks included in this phase
            - 0           # Reference number in task list below,
            - 1           # or absolute reference in Codabench database
        max_submissions: 1000
        max_submissions_per_day: 100
        execution_time_limit_ms: 60000
tasks:                    # Tasks for the above defined phase
    - index: 0
        name: 'Task a'    # For public display on leaderboad
        description: 'Dataset a'   # Private comments
        # Ingestion module:
        ingestion_program: ingestion_program.zip
        input_data: input_data_a.zip
        # Scoring module
        scoring_program: scoring_program.zip
        reference_data: reference_data_a.zip
        # whether the ingestion program is run first, then the
        # scoring program, or the are run in parallel
        ingestion_only_during_scoring: True
    - index: 1
        name: 'Task b'
        description: 'Dataset b'
        # Ingestion module:
        ingestion_program: ingestion_program.zip
        input_data: input_data_b.zip
        # Scoring module
        reference_data: reference_data_b.zip
        scoring_program: scoring_program.zip
        ingestion_only_during_scoring: True
leaderboards:               # Leader board form
    - title: Results        # single leaderboard supported in this version
        key: main           # main key, leave untouched
        columns:
        - title: 'Acc'      # Name of the column displayed
            key: acc        # Data key name used by scoring program
            index: 0        # Order of columns
            sorting: desc   # Sort in descending order
        - title: 'BalAcc'
            key: bacc
            index: 1
            sorting: desc
```

References

[1] Robert M Bell and Yehuda Koren. Lessons from the netflix prize challenge. *Acm Sigkdd Explorations Newsletter*, 9(2):75–79, 2007.

[2] Olga Russakovsky, Jia Deng, Hao Su, Jonathan Krause, Sanjeev Satheesh, Sean Ma, Zhiheng Huang, Andrej Karpathy, Aditya Khosla, Michael Bernstein, Alexander C. Berg, and Fei-Fei Li. Imagenet large scale visual recognition challenge. *International Journal of Computer Vision*, 115(3):211–252, 2015.
